# Whole-Exome Sequencing Reveals Recurrent but Heterogeneous Mutational Profiles in Sporadic WHO Grade 1 Meningiomas

**DOI:** 10.3389/fonc.2021.740782

**Published:** 2021-11-17

**Authors:** María González-Tablas, Carlos Prieto, Daniel Arandia, María Jara-Acevedo, Álvaro Otero, Daniel Pascual, Laura Ruíz, Iván Álvarez-Twose, Andrés Celestino García-Montero, Alberto Orfao, María Dolores Tabernero

**Affiliations:** ^1^ Instituto de Investigación Biomédica de Salamanca (IBSAL), University Hospital of Salamanca, Salamanca, Spain; ^2^ Centre for Cancer Research (Centro de Investigación del Cáncer de Salamanca (CIC)-Instituto de Biología Molecular y Celular del Cáncer (IBMCC), Centro Superior de Investigaciones Científicas (CSIC)/Universidad de Salamanca (USAL), IBSAL) and Department of Medicine, University of Salamanca, Salamanca, Spain; ^3^ Biomedical Research Networking Centre on Cancer– Centro de Investigación Biomédica en Red de Cáncer (CIBER-ONC) (CB16/12/00400), Instituto de Salud Carlos III, Madrid, Spain; ^4^ Bioinformatics Service Servicio de Apoyo a la Investigación de la Universidad de Salamanca (NUNCLEUS), University of Salamanca, Salamanca, Spain; ^5^ Neurosurgery Service, University Hospital of Salamanca, Salamanca, Spain; ^6^ Sequencing Service Servicio de Apoyo a la Investigación de la Universidad de Salamanca (NUNCLEUS), University of Salamanca, Salamanca, Spain; ^7^ Instituto de Estudios de Mastocitosis de Castilla La Mancha, Virgen del Valle Hospital, Toledo, Spain; ^8^ Spanish Network on Mastocytosis Red Española de Mastocitosis (REMA), Salamanca, Spain; ^9^ Spanish National DNA Bank Carlos III, University of Salamanca, Salamanca, Spain; ^10^ Instituto de Estudios de Ciencias de la Salud de Castilla y León (IECSCYL-IBSAL), Salamanca, Spain

**Keywords:** mutational profiles, whole exome sequencing (WES), *NF2*, *PTEN*, cytogenetics, WHO grade 1 meningioma

## Abstract

Human WHO grade 1 meningiomas are generally considered benign tumors; despite this, they account for ≈50% of all recurrent meningiomas. Currently, limited data exist about the mutational profiles of grade 1 meningiomas and patient outcome. We investigated the genetic variants present in 32 WHO grade 1 meningiomas using whole exome sequencing, and correlated gene mutational profiles with tumor cytogenetics and patient outcome. Overall, WHO grade 1 meningiomas harbored numerous and heterogeneous genetic variants, which most frequently affected the *NF2* (47%) gene and to a less extent the *PNMA6A* (22%), *TIGD1* (16%), *SMO* (13%), *PTEN* (13%), *CREG2* (9%), *EEF1A1* (6%), *POLR2A* (6%), *ARID1B* (3%), and *FAIM3* (3%) genes. Notably, non-synonymous genetic variants of *SMO* and *POLR2A* were restricted to diploid meningiomas, whereas *NF2* mutations were only found among tumors that showed -22/22q^─^ (with or without a complex karyotype). Based on *NF2* mutations and tumor cytogenetics, four genetic profiles were defined with an impact on patient recurrence-free survival (RFS). These included (1) two good-prognosis tumor subgroups—diploid meningiomas (n=9) and isolated -22/22q^─^ associated with *NF2* mutation (n=7)—with RFS rates at 10 y of 100%; and (2) two subgroups of poor-prognosis meningiomas—isolated -22/22q^─^ without *NF2* mutation (n=3) and tumors with complex karyotypes (n=11)—with a RFS rate at 10 y of 48% (p=0.003). Our results point out the existence of recurrent but heterogeneous mutational profiles in WHO grade 1 meningiomas which have an impact on patient outcome.

## Introduction

Sporadic meningioma is the most common primary brain tumor, among which WHO (World Health Organization) grade 1 (i.e., low grade/benign) meningiomas represent the great majority (≈85%) of cases (1). Compared to WHO grade 2 (atypical) and grade 3 (anaplastic) meningiomas, WHO grade 1 tumors typically show benign histopathological features associated with a better outcome (2). However, grade 1 meningiomas still show a heterogeneous clinical behavior (3). Thus, a smaller fraction of WHO grade 1 meningioma patients (earlier or later) show tumor recurrence, associated with a poorer outcome, such recurrent WHO grade 1 tumors accounting for ≈50% of all recurrent meningiomas (2). Despite the cytogenetic profile of grade 1 meningiomas has been extensively investigated in the past, and some chromosomal alterations (e.g., monosomy of chromosome 22) and cytogenetic profiles—e.g., del(1p) and monosomy 14—have been associated with the outcome of WHO grade 1 meningiomas (3), at present there are still limited data about the type and frequency of other genetic alterations (i.e., point mutations) in these tumors (4–6) and their potential impact on patient outcome (7).

Neurofibromin 2 (*NF2*) was the first gene to be associated with an increased risk to develop meningioma (8). *NF2* is a tumor suppressor gene located at chromosome 22q12.2, which encodes for the merlin protein, that is inactivated in a significant fraction of meningiomas by either gene mutation and/or deletion of the 22q chromosomal region—i.e., del(22q -) or monosomy 22 (9). Despite this, the potential involvement of *NF2* in the development of sporadic meningioma still remains controversial (10) because it is not a hallmark of these tumors. In contrast, while early clinical studies showed no clear association in meningiomas between *NF2* gene mutation and the WHO tumor grade (4, 11), recent reports showed a frequency of *NF2* mutation that varies in WHO grade 1 tumors between 20–35% *vs* 50–72% in grade 2/3 meningiomas (6, 12), and *NF2*-mutated meningiomas have been reported to display a poorer outcome to that of *NF2* wild-type tumors (12, 13). In line with these later findings, *in vitro* studies also indicate that *NF2*-mutated meningiomas are associated with a slower growth than *NF2* wild-type tumors (9).

Other numerous genetic alterations, including extensive chromosome losses and gains (14) together with mutations in genes other than *NF2*, have been recurrently reported in meningiomas (5, 11, 12, 15) (**Supplementary Table 1**). Thus, meningiomas frequently show chromosomal alterations that, apart from del(22q)/monosomy 22 (12 *vs* 42%), include del(1p), del(7p), del(19p), monosomy 10, and/or monosomy 14 (16). Likewise, mutations involving the *AKT1* (v-Akt murine thymoma viral oncogene homolog-1), *KLF4* (Kruppel-like factor 4), *PIK3CA* (phosphatidylinositol 3-kinase), *SMARCA4* (actin-dependent regulator of chromatin, subfamily A, member 4), and *SMO* (Smoothened) genes have also been recurrently reported at variable frequencies in sporadic meningioma (12, 14, 15), together with altered *ARID1B*, *BAP1*, *PTEN*, *TP53*, and *TRAF7* gene profiles, as revealed *via* gene panel (15) and a limited number of whole exome sequencing (WES) plus whole genome sequencing (WGS) (4, 11, 12, 15) studies. In these later studies, WES/WGS of relatively limited numbers of mostly grade 2/3 meningiomas have been investigated (n=5, 5, 11 and 66 tumors) (12, 17), in addition to a few grade 1 tumors (n=17, 20 and 39, respectively) (11, 12, 15).

## Material and Methods

### Patients and Samples

A total of 32 human WHO grade 1 meningioma samples from 31 patients—21 females and 10 males; median age of 66 years, range: 24 to 83 years—diagnosed with sporadic meningioma at the University Hospital of Salamanca (Spain) were retrospectively studied. At the moment of closing this study, nine patients had shown tumor recurrence after a median (range) follow-up of 7 years (2–21). Tumor samples corresponding to tissue not required for diagnosis (n=32) and paired blood samples from 3/32 patients were obtained after each donor had given his/her informed consent according to the Declaration of Helsinki and after its approval by the ethical committees. DNA was subsequently extracted from the tumor tissue and blood nucleated cells, immediately after the samples had been obtained, and stored at −80°C at the Spanish National DNA Bank Carlos III (University of Salamanca, Salamanca, Spain), until analyzed. The study was approved by the local ethics committee of the University Hospital of Salamanca (Salamanca, Spain).

Two WES datasets were additionally used in this study for the definition of the incidence of genetic variants in the general population: (i) one dataset included sequencing data from the Genome Aggregation Database (gnomAD; large-scale sequencing project from 125,748 unrelated individuals publicly available for the scientific community), and (ii) the second dataset included data on 129 unrelated Spanish subjects with similar gender (63F:66M) and age distribution (median 47 y; range 20–76 y) to the meningioma patient cohort—kindly provided by the Spanish Network on Mastocytosis (REMA)—to exclude variants that are commonly observed in the Spanish population.

### Next-Generation Sequencing of Meningioma DNA Samples

Genomic DNA was extracted from tumor tissues of 32 sporadic meningiomas, and blood DNA from three of the former patients, as previously described (16). DNA quantity and quality were assessed using the NanoDrop-1000 technology (Nano-Drop Technologies, Wilmington, DE, USA), and 100 ng of gDNA was used per sample to prepare individual sequencing libraries *via* the Kapa HyperPlus kit (Roche Diagnostics, Penzberg, Germany). For each library, DNA hybridization and capture were performed using the SeqCap EZ MedExome kit (Roche Diagnostics) with a global coverage of 47 Mb, including the coded regions identified in (i) the CCDS 17 RefSeq CDS (version from August 2014) available at www.ncbi.nlm.nih.gov/projects/CCDS, (ii) the Ensembl 76 CDS (filtered by biotype) available at www.ensembl.org/Homo_sapiens, (iii) the VEGA 56 CDS, (iv) the GENCODE 20 CDS (www.gencodegenes.org/human/release_20.html), and (v) the miRBase 21 (www.mirbase.org) publicly available databases. Sequencing was performed on an Illumina NovaSeq genome sequencer (Illumina, San Diego, CA, USA) with an average coverage of >130× per exome and a read length of 2×150 pb. FASTQ files containing WES data were obtained for each individually sequenced tumor and blood sample.

### Identification of Genetic Variants in Meningioma Samples

The Fastp software was used to check for the quality of sequencing data and to remove the remaining adaptamer sequences (18). Then, the resulting FASTQ files (with read pairs) were aligned to the GRCh38 human reference genome using the BWA (Burrows-Wheeler Aligner) software (http://bio-bwa.sourceforge.net/version 0.7.12). BWA-MEM algorithm was run with default parameters. Aligned files were then analyzed using the GATK software (version 3.5), following the best practices manual for variant calling (19). The Picard software (https://broadinstitute.github.io/picard/) was subsequently used to sort data, and duplicated reads were marked *via* the samtools (20). Quality of variants was recalibrated and insertions/deletions realigned using the GATK software. The *UnifiedGenotyper* method (GATK software) was used for variant calling. Additional filters described below were subsequently applied to discriminate between both acquired genetic variants (i.e., somatic mutations) and germline variants *vs* genetic variants present in the Spanish population—i.e., single nucleotide polymorphisms (SNP). The same data analysis protocol was applied for the tumor and blood DNA sequences from our series and the control (publicly available) datasets. Resulting variant calling files (VCF) were processed with SNPEffect (21) and Annovar (22), which append variant annotation information, functional effect prediction, and alternative allele frequencies (AAF) in the ExAC (23), 1000 Genomes (24), and gnomAD (25) project databases. The Linux commands to reproduce and replicate the analyses performed in this work are available upon request to the authors.

Identification of genetic variants was first focused on genes previously found to be mutated in meningiomas: *AKT1*, *ARID1B*, *BAP1*, *CDKN2A*, *CDKN2C*, *GNA11*, *KLF4*, *NF2*, *NRAS*, *PIK3CA*, *POLR2A*, *PTEN*, *SF3B1*, *SMARCA4*, *SMARCB1*, *SMO*, *TERT*, *TP53*, and *TRAF7*, and subsequently, in other genes that contained more mutations than found in the control dataset of the Spanish population. Cutoff values to define genetic variants were based on previous standard criteria: only those non-synonymous coding genetic variants were initially selected, and the sequences obtained were subsequently compared with our Spanish control and gnomAD population databases to discriminate between somatic variants and single-nucleotide polymorphisms. From all variants identified, only exonic variants with AAF ≥0.20 in the genomic regions sequenced which had not been previously reported in control Spanish, African, or European (non-Finnish) populations were considered in order to avoid complex genomic regions or low-quality variants. Moreover, only genes showing variants in >2 meningioma samples were further studied to avoid small size or rarely mutated genes. Filter values were adjusted based on *NF2* mutations, which were used as the gold standard positive. Finally, genes with at least five times more mutations than observed in the control dataset were manually curated to obtain a list of highly mutated genes.

For the identification of somatic genetic variants, somatic genomic mutation analyses were run up after all synonymous variants had been removed; variants with <40 reads were not considered. Six paired blood (n=3) and meningioma tumor samples (n=3) were used to confirm somatic mutations in the tumor of a subset of the patients that were missing in blood DNA. Variants detected in meningiomas at allele frequencies <0.01, match with the definition of rare variants and at least 10 reads validate the mutation, were considered to correspond to somatic mutations, while above this cutoff they could correspond to germinal mutations.

### Identification of Copy Number Alterations

Frozen tumor samples obtained after surgery were also assessed in parallel by interphase fluorescence *in situ* hybridization (iFISH) and/or single-nucleotide polymorphism (SNP) arrays, to identify numerical (copy number) alterations for 12 chromosomal regions scattered throughout the genome, which are frequently altered in meningiomas and for the (whole) 24 distinct human chromosomes, respectively. For iFISH analyses, a panel of probes directed against DNA sequences of 12 different chromosome regions frequently altered in meningiomas (1p36/1q25, 7, 9p34, 10, 14q32.3, 15q22, 17q21, 18q21, 22q11.2, X, and Y) obtained from Vysis Inc. (Abbott Laboratories, Santa Clara, CA, USA) were used in double stainings, as previously described (3). In turn, the high-density 500K GeneChip Mapping and the Genome-Wide Human SNP 6.0 Arrays (Affymetrix, Thermo-Fisher Scientific, Waltham, MA, USA) were used to determine the (cytogenetic) CNA profile of each individual meningioma, following the manufacturer’s instructions, as described elsewhere (16). SNP data are available at the GEO public database (access code: GSE42624).

### Statistical Analyses

For all statistical analyses, the SPSS software (SPSS 25.0, IBM SPSS, Armonk, NY, USA) was used. The Chi-square test and the Mann-Whitney U test were used to compare different groups of patients for categorical and continuous variables, respectively. Survival curves were plotted according to the Kaplan and Meier method, and the (two-sided) log-rank test was used to assess the statistical significance of differences in recurrence-free survival (RFS; defined from tumor diagnostic surgery to disease recurrence, by imaging techniques) between distinct groups of patients. RFS data were available in 30 meningioma patients with a median follow-up of 8 years, range 1 to 21 years, after first surgery.

## Results

### Genetic Profile of WHO Grade 1 Meningiomas

*NF2* (15/32 tumors, 47%) gene was the most frequently showed non-synonymous and/or synonymous genetic variants among WHO grade 1 meningiomas (**Table 1** and **Supplementary Table 1**). Other genes that recurrently carried non-synonymous genetic variants at lower frequencies included the *PNMA6A* (7/32 tumors, 22%), *TIGD1* (5/32, 16%), *SMO* (4/32, 13%), *PTEN* (4/32, 13%), *CREG2* (3/32, 9%), *EEF1A1* (2/32, 6%), and *POLR2A* (2/32, 6%) genes; in turn, the *ARID1B* and *FAIM3* genes were found to be mutated in a single tumor each (1/32, 3%) (**Table 1**). From these later nine genes, *TIGD1* was the only gene for which synonymous genetic variants were also detected (in addition to non-synonymous variants) in 5/32 tumors (16%). Out of all recurrent non-synonymous genetic variants identified in our patients, only a subset of them involving the *ARID1B*, *NF2*, *POLR2A*, *PTEN*, *SMARCA4*, and *SMO* genes had been previously reported in sporadic meningiomas (**Supplementary Table 2**).

**Table 1:** Synonymous and non-synonymous genetic variants detected in WHO grade 1 meningiomas (n = 32) ordered by the frequency of tumors with non-synonymous genetic variants identified.

Gene	Synonymous		Non-synonymous	
	N. of genetic variants	N. of tumors with genetic variants (%)	N. of genetic variants	N. of tumors with genetic variants (%)
*NF2*	0	0/32 (0%)	16	15/32 (47%)
*PNMA6A*	0	0/32 (0%)	1	7/32 (22%)
*TIGD1*	2	5/32 (16%)	5	5/32 (16%)
*PTEN*	0	0/32 (0%)	1	4/32 (13%)
*SMO*	0	0/32 (0%)	2	4/32 (13%)
*CREG2*	0	0/32 (0%)	2	3/32 (9%)
*EEF1A1*	0	0/32 (0%)	2	2/32 (6%)
*POLR2A*	0	0/32 (0%)	1	2/32 (6%)
*ARID1B*	0	0/32 (0%)	1	1/32 (3%)
*FAIM3*	0	0/32 (0%)	1	1/32 (3%)
*SMARCA4*	2	2/32 (6%)	0	0/32 (0%)
Total	4	7/32 (22%)	32	27/32 (84%)

**Figure 1:**
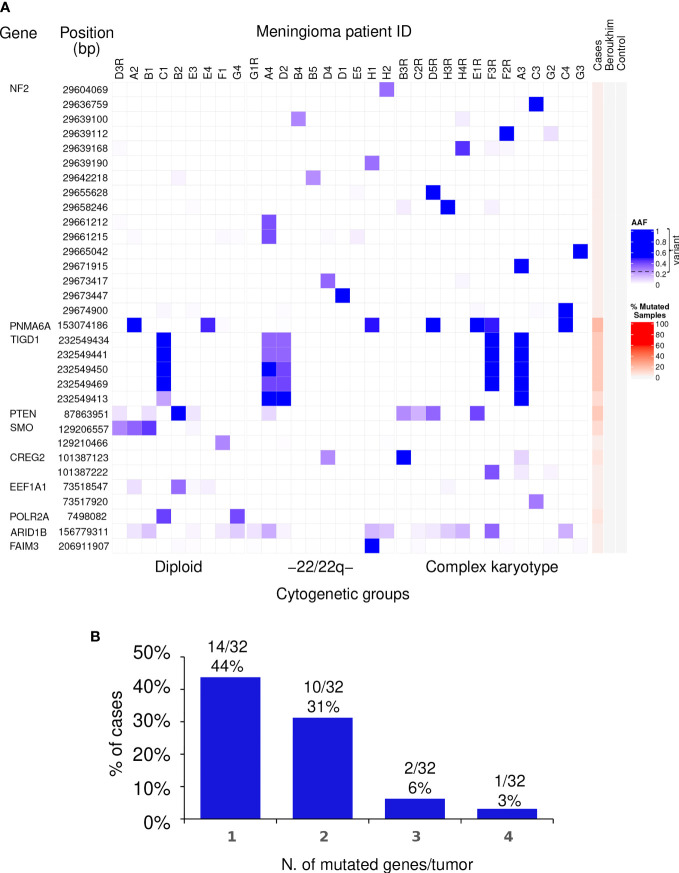
Distribution of non-synonymous genetic variants found altered in WHO grade 1 meningiomas. **(A)** Heat map of the non-synonymous genetic variants identified (rows) in individual WHO grade 1 meningiomas (columns) classified according to their chromosomal copy number cytogenetic profile. For each genetic variant, alternative allele frequencies (AAF) are color coded in blue from the lowest (light blue) to the highest (dark blue) percent values using the 0.25 AAF value as the cutoff for variant detection. Patients that showed tumor recurrence are identified on top of each column by the patient ID code followed or not by the letter “R” for the recurrent *vs* non-recurrent tumors. The red color scale represents the overall percentage of mutated samples among the WHO grade 1 meningiomas studied in our cohort as well in the controls. **(B)** The number and frequency of tumors carrying non-synonymous genetic variants in different (numbers of) genes are displayed.

In more detail, a total of 16 different non-synonymous variants of the *NF2* gene for which no synonymous variants were found, were identified in 15/32 tumors (47%), one of which displayed two different variants (**Table 1** and **Figure 1**). Of note, all 16 genetic variants of the *NF2* gene identified affected the FERM domain of the NF2-coded merlin protein with potential (predicted) impact at the functional protein level (**Supplementary Figure 1**).

In contrast to *NF2*, recurrent non-synonymous genetic variants were observed for a total of six other genes, i.e., *PNMA6A*, *TIGD1*, *PTEN*, *SMO*, *CREG2*, and *POLR2A*, with the following distribution (**Figure 1A**): (i) a single recurrent non-synonymous genetic variant of the *PNMA6A* gene at 153074186bp was identified in 7/32 (22%) meningiomas; (ii) the same four *TIGD1* variants were identified in 5/32 (16%) meningiomas at 232549434bp, 232549441bp, 232549450bp, and 232549469bp, four of these five tumors showing an additional genetic variant of this gene located at 232549413bp, all five non-synonymous genetic variants of the *TIGD1* gene being located along a relatively short region of 36bp, from the 232549413bp to the 232549469bp position (**Figure 1A**); (iii) a single non-synonymous variant of *PTEN* at the 87863951bp position was identified in 4/32 tumors (13%); (iv) one non-synonymous genetic variant of the *SMO* gene at 129206557bp was detected in 3/32 (9%) patients; (v) one *CREG2* variant at 101387123bp was identified in another 2/32 tumors (6%); and finally, (vi) one *POLR2A* variant at 7498082bp was detected in 2/32 (6%) meningiomas (**Figure 1A**). Overall, non-synonymous genetic variants in ≥1 gene were found in 27/32 WHO grade 1 meningiomas, the great majority (24/32; 75%) of them showing one or two altered genes, three in two tumors (6%), and ≥4 genes were found to carry non-synonymous genetic variants in one patient (3%) (**Figure 1B**).

In a subset of 3/32 patients, mutational analyses were performed in paired blood and primary tumor samples. One genetic variant involving the *PNMA6A* gene was present in 1/3 meningiomas investigated. In turn, non-synonymous variants of the *NF2*, *POLR2A*, and *PTEN* genes were identified in tumor samples from 2/3, 1/3, and 1/3 patients investigated, respectively, while they were systematically absent in blood DNA (**Supplementary Table 3**), supporting the specific acquisition of these genetic variants in the tumor cells of these patients.

### Frequency of Non-Synonymous Genetic Variants in Different Cytogenetic Subgroups of WHO Grade 1 Meningiomas

From the cytogenetic point of view, our 32 WHO grade 1 meningiomas were divided into three groups based on their chromosome copy number status by iFISH and/or SNP-arrays: (i) diploid tumors (meningiomas that showed no chromosome losses and/or gains), 9 cases (28%); (ii) meningiomas with an isolated loss of chromosome 22/del(22q), 10 tumors (31%); and, (iii) meningiomas with complex karyotypes consisting of losses and/or gains involving ≥2 chromosomes/chromosome regions, 13 cases (41%) (**Table 2**). Overall, the mean number of altered genes and/or the mean ( ± 1SD) number of non-synonymous genetic variants per tumor slightly increased (p>0.05) from diploid tumors (1.2 ± 0.7 altered genes/tumor and 1.7 ± 1.5 variants/tumor) and meningiomas carrying isolated monosomy 22/22q^─^ (1.2 ± 0.9 altered genes/tumor and 2.1 ± 2.3 genetic variants/tumor), to cases with complex karyotypes (1.6 ± 1.0 altered genes/tumor and 2.1 ± 1.9 genetic variants/tumor) (**Table 2**). In turn, the frequency of non-synonymous genetic variants that affected individual genes in these three cytogenetic subgroups of meningiomas revealed different mutational profiles among them for the *NF2*, *SMO*, and *POLR2A* genes (**Table 2**). Thus, *NF2* mutations were restricted to the majority of tumors displaying monosomy 22/22q^─^ (70%) and complex karyotypes (62%)—all such cases with complex karyotypes plus *NF2* mutation including monosomy 22/22q^─^ —while absent (0%) in diploid meningiomas (p= 0.001) (**Table 2**); in contrast, *SMO* and *POLR2A* genetic variants were restricted to diploid meningiomas—44 and 22% cases *vs* 0% among tumors with isolated monosomy 22/22q^─^ and complex karyotypes (p=0.001 and p=0.02, respectively)—and *PTEN* variants were exclusively found in meningiomas that had complex and diploid karyotypes (23 and 11%, respectively), while absent in tumors with an isolated monosomy 22/22q^─^ (p=0.15) (**Table 2**).

**Table 2:** Frequency of non-synonymous genetic variants in different cytogenetic subtypes of WHO grade 1 meningiomas as defined by their chromosome copy number profile.

Gene	Genetic subgroup of WHO grade 1 meningiomas			p-value
	Diploid (n = 9)	Monosomy 22/22q^─^ (n = 10)	Complex karyotype (n = 13)	
	N. of variants/N. of tumors (%)	N. of variants/N. of tumors (%)	N. of variants/N. of tumors (%)	
*NF2*	0/0 (0%)	8/7 (70%)^a^	8/8 (62%)	0.001^*^
*PNMA6A*	1/2 (22%)	1/1 (10%)	1/4 (30%)	0.49
*PTEN*	1/1 (11%)	0/0 (0%)	1/3 (23%)	0.15
*TIGD1*	5/1 (11%)	10/2 (20%)	9/2 (15%)	0.88
*SMO*	2/4 (44%)	0/0 (0%)	0/0 (0%)	0.001^*^
*POLR2A*	1/2 (22%)	0/0 (0%)	0/0 (0%)	0.02^*^
*EEF1A1*	1/1 (11%)	0/0 (0%)	1/1 (8%)	0.58
*CREG2*	0/0 (0%)	1/1 (10%)	2/2 (15%)	0.47
*FAIM3*	0/0 (0%)	1/1 (10%)	0/0 (0%)	0.32
*ARID1B*	0/0 (0%)	0/0 (0%)	1/1 (8%)	0.47
N. of mutated genes/tumor	1.2 ± 0.7	1.2 ± 0.9	1.6 ± 1.0	0.63
N. of variants/tumor	1.7 ± 1.5	2.1 ± 2.3	2.1 ± 1.9	0.67

Results expressed as number (percentage) or as mean values ± one standard deviation (SD); ^a^one meningioma had two different NF2 variants; *statistically significant differences detected between diploid tumors and the other two cytogenetic groups of meningioma.

### Non-Synonymous Genetic Variants in Recurrent *vs* Non-Recurrent WHO Grade 1 Meningiomas

**Figure 2:**
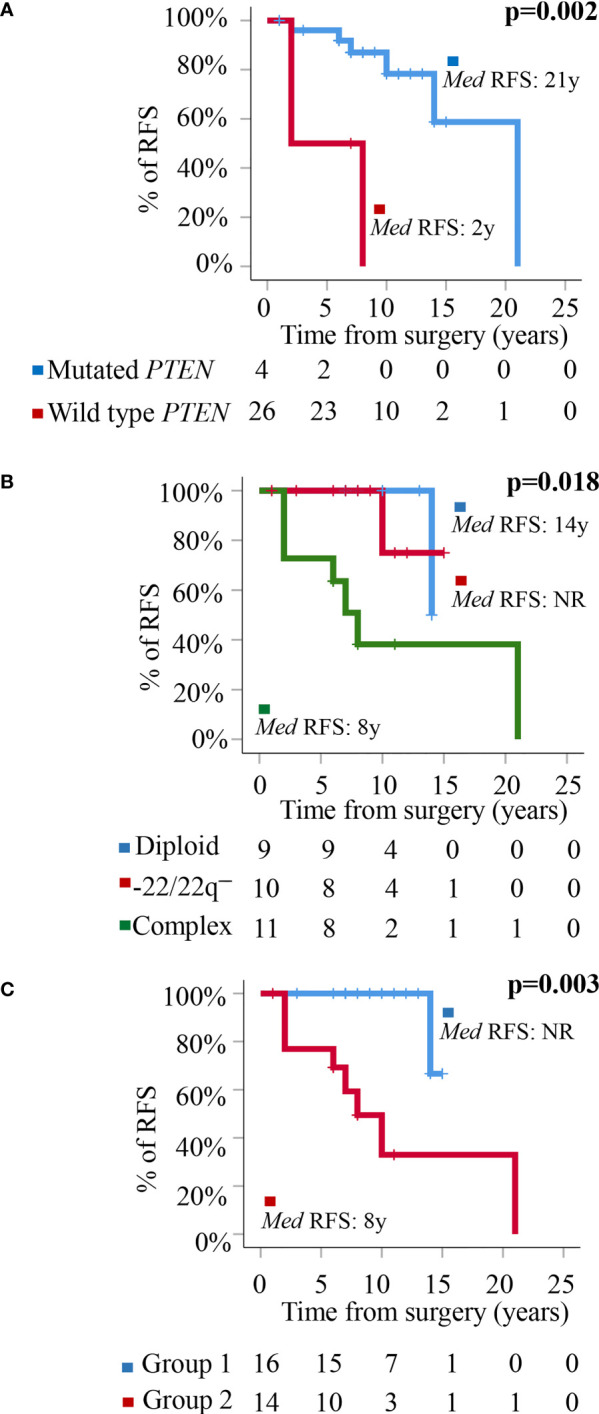
Prognostic impact of *PTEN* mutation and the cytogenetic and molecular tumor profiles in the outcome. **(A)** Prognostic impact of the presence of genetic variants of *PTEN* on patient relapse-free survival (RFS). **(B)** Prognostic impact of the cytogenetic profile of WHO grade 1 meningiomas on patient RFS. **(C)** Prognostic impact of the overall cytogenetic and mutational profile on RFS of WHO grade 1 meningiomas distributed into a good-prognosis group (Group 1: meningiomas with a diploid karyotype and isolated monosomy 22/22q^─^ associated with *NF2* mutation) (n = 16) and a poor-prognosis group (Group 2: meningiomas with isolated monosomy 22/22q^─^ in the absence of *NF2* mutations and tumors with a complex karyotype associated or not with mutation of the *NF2* gene) (n = 14).

At the moment of closing this study, 10/32 WHO grade 1 tumors had shown recurrence of the disease. These included 1/9 (11%) diploid meningiomas, 1/10 (10%) tumors carrying an isolated monosomy 22/22q^─^, and 8/13 (62%) meningiomas with complex karyotypes (p=0.009). Overall, a high frequency of tumors carrying non-synonymous genetic variants of the *PTEN* gene (all located at the 87863951pb chromosomal region) were found in recurrent *vs* non-recurrent tumors (30 *vs* 5%, p=0.04) (**Table 3**) in association with a poorer outcome (p=0.002)—recurrence-free survival (RFS) rate at 10 years of 85% (95% confidence interval: 68–100%) for *PTEN* mutated patients *vs* not reached for *PTEN* wild-type tumors—(**Figure 2A**). In contrast, no significantly different (p>0.05) frequencies of genetic variants involving the other altered *NF2*, *PNMA6A*, *CREG2*, *ARID1B*, *NF2*, *TIGD1*, *SMO*, *EEF1A1*, *POLR2A*, and *FAIM3* genes were observed in recurrent *vs* non-recurrent tumors (**Table 3**). In addition to the *PTEN* gene status, the overall cytogenetic profile of WHO grade 1 meningiomas also showed an impact (p=0.018) on RFS (**Figure 2B**). Based on the cytogenetic profile (diploid *vs* isolated monosomy 22/22q^─^ *vs* complex karyotype) and the presence *vs* absence of *NF2* gene mutations, four distinct genetic subgroups were defined, which could be pooled in two groups with significantly different RFS rates at 10 years (p=0.003): (i) a good-prognosis group (n=16) with median ( ± 95% confidence interval) RFS at 10 y of 100% (100%) consisting of diploid tumors (n=9) and meningiomas with isolated monosomy 22/22q^─^ associated with *NF2* mutation (n=7), and (ii) poor-prognosis meningiomas (n=14) with isolated monosomy 22/22q^─^ in the absence of *NF2* mutation (n=3) and complex karyotypes (n=11), with median ±95% confidence interval RFS at 10 y of 48% (19–77%) (**Figure 2C**).

**Table 3:** Distribution of the non-synonymous genetic variants identified in non-recurrent *vs* recurrent WHO grade 1 meningiomas.

Gene	Non-recurrent meningioma (n = 22)		Recurrent meningioma (n = 10)		p-value (X^2^)
	N. of variants/Total	N. of altered tumors (%)	N. of variants/Total	N. of altered tumors (%)	
*NF2*	11/12	11 (50%)	4/4	4 (40%)	0.59
*PTEN*	1/1	1 (5%)	1/3	3 (30%)	0.04
*SMO*	2/3	3 (14%)	1/1	1 (10%)	0.77
*PNMA6A*	1/4	4 (18%)	1/3	3 (30%)	0.45
*CREG2*	1/1	1 (5%)	2/2	2 (20%)	0.16
*TIGD1*	5/19	4 (18%)	4/4	1 (10%)	0.56
*ARID1B*	0	0 (0%)	1/1	1 (10%)	0.13
*EEF1A1*	3/3	2 (9%)	0	0 (0%)	0.33
*POLR2A*	1/2	2 (9%)	0	0 (0%)	0.33
*FAIM3*	1/1	1 (5%)	0	0 (0%)	0.49

Results expressed as number of variants/total number of variants and as number of tumors (percentage).

## Discussion

Previous studies have shown multiple genes and chromosomes to be recurrently altered in meningiomas (26). Among all altered genes, *NF2* is by far the most frequently mutated (0–46%) in the literature (4, 6, 8, 9, 12, 15, 27–29), followed by the *TRAF7* (2–38%) (4, 12, 13, 30)*, AKT1* (8–31%) (6, 7, 12, 13, 15, 31, 32), *KLF4* (9–15%; 100% of secretory meningiomas) (12, 13, 30, 32), *SMO* (6–32%) (7, 10, 12, 15), *TERT* (2–14%) (10, 33–38), and *PIK3CA* (0–7%) (10, 12, 39) genes, and other less studied genes, such as the *ARID1B*, *POLR2A*, and *PTEN* genes found to be mutated in 22, 6, and 1% of meningiomas, respectively (4, 5, 40). Typically, all above genes are recurrently altered in meningiomas across all WHO grades, despite controversial results have been reported about a greater frequency of the *NF2*, *KLF4*, and *SMO* gene variants in WHO grade 2/3 *vs* WHO grade 1 meningiomas (12, 15).

Despite all the above, so far, only a few studies (4, 11, 12) have applied NGS to investigate the presence of genetic variants across the whole exome/genome of meningiomas, usually in a series of patients that also included other CNS tumors (e.g., gliomas) (41) and/or that were preferentially focused on WHO grade 2/3 meningiomas (11). Thus, only a limited number of studies specifically included WES/WGS data on WHO grade 1 meningiomas for a total of 76 WHO grade 1 tumors with available data reported in the literature (n=17, 20, and 39) (11, 12, 15). Here we used WES to search for those genes more frequently altered in WHO grade 1 meningiomas and investigate the potential association between their alterations and both tumor cytogenetics and patient outcome.

Overall, WES revealed recurrent but heterogeneous mutational profiles in WHO grade 1 meningiomas with up to 10 genes showing non-synonymous genetic variants in at least one tumor, some of which were closely associated with the cytogenetic (i.e., chromosomal) tumor profiles. Thus, from those 9/10 genes found to be altered in ≥2 grade 1 meningiomas, the *NF2* gene was the most frequently carried non-synonymous genetic variants, in combination or not with other synonymous genetic variants, respectively. Another four genes (*PNMA6A*, *TIGD1*, *PTEN*, and *SMO*) were found to be altered in >10% of all WHO grade 1 meningiomas investigated, while the remaining *CREG2*, *EEF1A1*, and *POLR2A* genes were recurrently altered in a minority (<10%) of cases. Of note, paired analysis of blood and meningioma tissue samples: in a subset of three patients, both samples were available, revealing the presence of high-alternative allele frequencies (range: 96 to 98%) in both tissues for the *PNMA6A* non-synonymous gene variants identified, suggesting they might correspond to germinal variants of this gene. In contrast, all genetic variants of the *NF2*, *POLR2A*, and *PTEN* genes found in these three patients in which paired tumor and blood DNA were studied, and were absent in blood DNA and restricted to the tumor cell DNA where they were detected at frequencies ≤40%—except for NF2 tumors with -22/del(22q), suggesting these later variants would correspond to tumor-specific non-synonymous genetic variants, e.g., somatic mutations. However, further studies in which paired blood and tumor samples from larger series of meningioma patients are studied are necessary to confirm these preliminary observations. Overall, these results confirm the high frequency of genetic variants of the *NF2* gene and to a less extent also of the *PTEN*, *SMO*, and *POLR2A* genes, in WHO grade 1 meningiomas. At the same time we report here for the first time a relatively high frequency of genetic variants of the *PNMA6A* and *TIGD1* genes, which were absent in other series of healthy subjects and patients investigated in our laboratory (42–44). In contrast, we could not find in our WHO grade 1 tumors and control populations, non-synonymous genetic variants, for other genes previously reported to be recurrently mutated in meningiomas such as the *TRAF7*, *AKT1*, *KLF4*, *TERT*, and *PIK3CA* genes (10, 12, 39). Further investigations are thereby required to better understand the reason for these differences and the potential role of these later genes in meningioma.

Up to 80 different *NF2* mutations have been previously reported in meningiomas (4, 6, 8, 9, 11, 12, 15, 26–29). In line with these findings, 16 different non-recurrent non-synonymous variants of the *NF2* gene coded at chromosome 22q12.2 were observed in almost half of our WHO grade 1 meningiomas, in close association with monosomy 22/22q^─^, either as an isolated chromosomal alteration or in the context of a complex karyotype. Of note, only a quarter of all *NF2* variants found (genomic position and bp change: 29604069, c.71_72insA; 29639100, c.A251C; 29642218, c.T380G; 29661212, c.683delG) had been previously identified in meningiomas (5, 12, 27), while the other 12 are reported here for the first time. Lack of recurrent *NF2* mutations among our WHO grade 1 meningiomas, as well as in most series reported in the literature, suggests that sporadic meningioma cells frequently acquire *NF2* mutations randomly (11), in both WHO grade 1 and higher-grade tumors (9, 12). Despite such high variability on the specific *NF2* mutations found among our WHO grade1 meningiomas, *in silico* analysis revealed a common effect at the merlin protein level. Thus, all 16 genetic variants identified would target the FERM functional domain of the *NF2*-coded merlin protein. These results support the notion that despite *NF2* mutations are heterogenous, they all lead to inactivation of the merlin protein in the absence of wild-type *NF2*, and support a relevant role for the *NF2* gene in the oncogenesis of a significant fraction (≈50%) of WHO grade 1 meningiomas (11). In line with this hypothesis and previous literature data (9, 11), we found a close association between *NF2* mutation and monosomy 22/22q^─^, as all *NF2-*mutated tumors also carried monosomy 22/22q ^-,^ in association or not with a complex karyotype. In contrast with previous studies, mutations in genes other than *NF2*, which are coded in the vicinity of the *NF2* gene at chromosome 22q (e.g., the *SMARCB1* gene), were not detected in our patients, suggesting that mutations in these genes (12) are absent or rare (≤5%) in WHO grade 1 meningiomas, and thereby, they would play a limited role in the pathogenesis of WHO grade 1 meningiomas, in contrast with *NF2* gene mutations.

Other genes that were recurrently mutated in our series included the *PNMA6A*, *TIGD1*, *SMO*, *PTEN*, *CREG2*, *EEF1A1*, and *POLR2A* genes. Interestingly, some of these genes (*SMO* and *POLR2A*) were restricted to *NF2*-wild-type diploid tumors, or they (*PTEN*) predominated in *NF2-*wild-type cases. In turn, recurrent mutations in the *TIGD1* gene were found across all cytogenetic subgroups of meningiomas including both *NF2-*mutated and wild-type tumors. These results suggest that some of these later altered genes (e.g., *SMO*, *POLR2A*) might act as driver genes in a significant fraction of *NF2*-wild-type WHO grade 1 meningiomas (12, 15, 39), further studies being required to confirm this hypothesis.

When individually considered, only *PTEN* mutations together with the cytogenetic tumor profile showed a significant impact on patient outcome (i.e., RFS). These results confirm previous data from our group (3) indicating that complex karyotypes within WHO grade 1 meningiomas are associated with an increased risk of relapse. In turn, here we show for the first time an adverse prognostic impact for *PTEN* mutation, although most *PTEN*-mutated cases also carried a complex karyotype. In line with these findings, some authors have recently reported that the *PTEN* gene might play a critical role in regulating chromosome segregation to prevent gross genomic instability *via* regulation of the mitotic arrest deficient 2 (*MAD2*) gene (45); these finding might contribute to explain the association here reported between mutated *PTEN* and both a complex karyotype and a poorer outcome. Of note, this is the first study in which an association between *PTEN* gene mutations and a poor outcome is reported among WHO grade 1 meningiomas, similarly to what has been reported in tumor types other than meningioma, including glioma (46). However, these findings are in contrast with previous studies that found a similar frequency of *PTEN* mutations in meningiomas to the one here reported, but no association with RFS (47). Most interestingly, when we simultaneously considered the tumor cytogenetic patterns and the *NF2* mutation profile, four distinct genetic profiles were identified, which could be grouped into two good-prognosis genetic profiles (diploid karyotype and isolated monosomy 22/22q^─^ associated with *NF2* mutation), and two poor-prognosis genetic patterns (isolated monosomy 22/22q^─^ in the absence of *NF2* mutation and complex karyotypes) with significantly different RFS rates. Altogether, these results suggest that presence of *NF2* mutation in the context of an isolated monosomy 22/22q^─^ confer a low risk of recurrence for WHO grade 1 meningiomas, while complex karyotypes and isolated loss of chromosome 22 in the absence of *NF2* mutation would confer a poorer prognosis. So far, few studies have combined cytogenetic and molecular data for risk-stratification of WHO grade 1 and also grade 2/3 meningiomas. Therefore, our preliminary results on the prognostic impact of the above described molecular/genetic profiles still need to be confirmed in larger prospective series of WHO grade 1 meningiomas followed for long periods of time (11).

In summary, our results based on WES of WHO grade 1 meningiomas reveal recurrent but heterogeneous mutational and cytogenetic profiles, which might reflect different oncogenic pathways associated with distinct patterns of chromosomal instability and patient outcome. Further studies in larger series of both WHO grade 1 and grade 2/3 meningiomas are required to confirm these observations.

## Data Availability Statement

The datasets presented in this study can be found in online repositories. Genomic data analyzed in this study is available at EBI ENA under the PRJEB43143 project identifier.

## Ethics Statement

The studies involving human participants were reviewed and approved by the local ethics committee of the University Hospital of Salamanca (Salamanca, Spain). The patients/participants provided their written informed consent to participate in this study.

## Author Contributions

All authors have contributed significantly to this article: AOr and MT contributed to the study design. DA, AOt, DP, LR, IÁ-T, and AG-M contributed to the collection of samples and clinical data. M-GT, CP, MJ-A, and MT developed the methodology and analysis. Finally, MG-T, CP, AOr, and MT contributed to the original draft preparation. All authors contributed to the article and approved the submitted version.

## Funding

This work was supported by grants: IBY 17/00002 from IBSAL (Salamanca, Spain), GRS2132/A2020 from Junta de Castilla y León (Spain), and CB16/12/00400 from CIBER-ONC and FONDOS FEDER (Instituto de Salud Carlos III, Ministerio de Economía y Competitividad, Madrid, Spain).

## Conflict of Interest

The authors declare that the research was conducted in the absence of any commercial or financial relationships that could be construed as a potential conflict of interest.

## Publisher’s Note

All claims expressed in this article are solely those of the authors and do not necessarily represent those of their affiliated organizations, or those of the publisher, the editors and the reviewers. Any product that may be evaluated in this article, or claim that may be made by its manufacturer, is not guaranteed or endorsed by the publisher.

## Abbreviations

AAF, alternative allele frequencies; Bp, pair base; CAN, copy number alterations; Del, deletion; FERM, Four-point-one, Ezrin, Radixin, Moesin; iFISH, fluorescence *in situ* hybridization; gnomAD, Genome Aggregation Database; NGS, next-generation sequencing; NRAS, NRAS Proto-Oncogene; GTPase; REMA, Spanish Network on Mastocytosis; SNP, nucleotide polymorphism; RFS, recurrence-free survival; WES, whole exome sequencing; WGS, whole genome sequencing; WHO, World Health Organization.
