# Supplementary material for: Whole-Exome Sequencing Reveals Recurrent but Heterogeneous Mutational Profiles in Sporadic WHO Grade 1 Meningiomas
Source: Front Oncol. 2021 Nov 17;11:740782. doi: 10.3389/fonc.2021.740782 (PMC8635692; doi:10.3389/fonc.2021.740782)
Supplement: Supplementary file 1 [file DataSheet_1.docx]

Supplementary Material

# Supplementary Figures and Tables

## Supplementary Tables

**Supplementary Table 1.** Detailed description of the specific non-synonymous genetic variants identified in WHO grade 1 meningiomas (n=32).

| *Gene* | Variant location | cDNA base position | Affected aminoacid | AF in gnomAD | AAF in our series | Recurrent variants |
| --- | --- | --- | --- | --- | --- | --- |
| *ARID1B* | 156779311 | c.G1382C | p.G461A | 0 | 0.33 | No |
| *CREG2* | 101387123 | c.A335T | p.Q112L | 0 | 0.25-0.57 | Yes |
|  | 101387222 | c.A236T | p.H79L | 0 | 0.38 | No |
| *EEF1A1* | 73517920 | c.C1279T | p.R427C | 0 | 0.29 | No |
|  | 73518547 | c.C836T | p.T279I | 0.00002 | 0.31 | No |
| *FAIM3* | 206911907 | c.C197A | p.S66Y | 0 | 0.51 | No |
| *NF2* | 29604069 | c.71_72insA | p.V24fs | 0.00003 | 0.32 | No |
|  | 29636759 | c.124delA | p.K42fs | 0 | 0.57 | No |
|  | 29639100 | c.A251C | p.H84P | 0.00001 | 0.26 | No |
|  | 29639112 | c.264delG | p.K88fs | 0.000004 | 0.50 | No |
|  | 29639168 | c.G319T | p.E107X | 0 | 0.44 | No |
|  | 29639190 | c.342delA | p.T114fs | 0 | 0.31 | No |
|  | 29642218 | c.T380G | p.L127X | 0 | 0.25 | No |
|  | 29655628 | c.G511A | p.W184X | 0 | 0.71 | No |
|  | 29658246 | c.658delA | p.N220fs | 0 | 0.85 | No |
|  | 29661212 | c.683delG | p.K228fs | 0.000008 | 0.38 | No |
|  | 29661215 | c.G686T | p.G229V | 0 | 0.39 | No |
|  | 29665042 | c.C863G | p.S288X | 0 | 0.69 | No |
|  | 29671915 | c.1090-1091insA | p.M363fs | 0 | 0.50 | No |
|  | 29673417 | c.1272delC | p.R424fs | 0.00003 | 0.33 | No |
|  | 29673447 | c.1302delG | p.E434fs | 0 | 0.77 | No |
|  | 29674900 | c.A1405T | p.K469X | 0 | 0.64 | No |
| *PNMA6A* | 153074186 | c.T1124G | p.L375R | 0 | 0.46-0.98 | Yes |
| *POLR2A* | 7498082 | c.C1207A | p.Q403K | 0 | 0.39-0.41 | Yes |
| *PTEN* | 87863951 | c.T2A | p.L1Q | 0 | 0.25-0.5 | Yes |
| *SMO* | 129206557 | c.C1234T | p.L412F | 0 | 0.26-0.43 | Yes |
|  | 129210466 | c.G1570C | p.A524P | 0 | 0.26 | No |
| *TIGD1* | 232549413 | c.C470T | p.A157V | 0 | 0.5-1 | Yes |
|  | 232549434 | c.C449T | p.A150V | 0 | 0.33-0.6 | Yes |
|  | 232549441 | c.A442G | p.I148V | 0.00001 | 0.33-0.6 | Yes |
|  | 232549450 | c.T433C | p.F145L | 0 | 0.4-0.6 | Yes |
|  | 232549469 | c.G414C | p.R138S | 0 | 0.4-1 | Yes |

AF: Allele frequency; AAF: Alternative allele frequency detected in each individual patient or its range in case of recurrent variant; ^a^: Variant found in whole genomic studies.

**Supplementary Table 2**. Recurrent genetic variants previously reported in sporadic meningiomas according to their WHO tumor grade.

| Mapped gene ID | Chromosome location | Variant | Frequency of mutated tumors (N. of tumors and WHO grade) | Reference |
| --- | --- | --- | --- | --- |
| *AKT1/207* | 14q32.33 | E17K | 11% WHO 1 (47 WHO 1, 15 WHO 2 & 3 WHO 3) | (15) |
|  |  |  | 9% WHO 1, 9% WHO 2 (39 WHO I & 11 WHO 2) | (12) |
|  |  |  | 8% WHO 1, 3% WHO 2 (705 WHO 1, 144 WHO 2 & 96 WHO 3) | (31) |
|  |  |  | 12% WHO I, 44% WHO 2 (69 WHO I & 9 WHO 2 **olfactory** groove meningiomas) | (7) |
|  |  |  | 29% (5 WHO 1 & 8 WHO 2 & 1 WHO 3) | (6) |
|  |  |  | 31% (82 WHO 1 &11 WHO 2) | (32) |
|  |  |  | 8% (84 WHO 1 & 6 WHO 2 & 2 WHO 3) | (13) |
| *APC/324* | 5q22.2 | V1943I | 6% (15 WHO 1 & 3 WHO 2) | (10) |
| *ARID1B/57492* | 6q25.3 | A1702T  R1977Q | 22% (9 WHO 3) | (4) |
| *BAP1/8314* | 3p21.1 | G220_splice  Y173X | 7% (5 WHO 1 & 8 WHO 2 & 1 WHO 3) | (6) |
| *EPB41L3/23136* | 18p11.31 | A555T  T950K | 2% (83) | (48) |
| *FBXW7/55294* | 4q31.3 | G246X  N365fs | 14% (5 WHO 1 & 8 WHO 2 & 1 WHO 3) | (6) |
| *GABRA6/2559* | 5q34 | V269I | 6% (15 WHO 1 & 3 WHO 2) | (10) |
| *GSE1/23199* | 16q24.1 | S724L  S755L  S828L | 11% (15 WHO 1 & 3 WHO 2) | (10) |
| *KDR/3791* | 4q12 | E815Q | 7% (15 WHO 1 & 3 WHO 2) | (10) |
| *KLF4/8314* | 9q31.2 | K409Q | 15% WHO I (34 WHO 1 &11 WHO 2)  (3/3 100% **secretory** meningiomas) | (12) |
|  |  |  | 12% (82 WHO 1 & 11 WHO 2) | (32) |
|  |  |  | 9% (84 WHO 1 & 6 WHO 2 &2 WHO 3) | (13) |
|  |  |  | 100% (16 **secretory** meningiomas) | (30) |
| *MLLT10/8028* | 10p12.31 | ^a^rs11012732-A | 87% (961) | (49) |
| *MN1/4330* | 22q12.1 | P540insQ  P522insQ  P972S  P1138R | 22% (9 WHO 3) | (4) |
| *MTOR/2475* | 1p36.22 | D1279V | 2% WHO I (47 WHO 1, 15 WHO 2 & 3 WHO 3) | (15) |
| *MUC2/4583* | 11p15.5 | P1864S | 11% (9 WHO 3) | (4) |
| *NF2/4771* | 22q12.2 | ^b^79 mutations | 50% (5 WHO 1 & 8 WHO 2 & 1 WHO 3) | (6) |
|  |  |  | 38% (14) | (8) |
|  |  |  | 13% (80) | (29) |
|  |  |  | 45% (42) | (28) |
|  |  |  | 47% (100) | (27) |
|  |  |  | 30% (20) | (9) |
|  |  |  | 41% WHO 1, 73% WHO 2 (34 WHO 1 & 11 WHO 2) | (12) |
|  |  |  | 40% WHO 1, 60% WHO 2 (47 WHO I, 15 WHO 2 & 3 WHO 3) | (15) |
|  |  |  | 11% (9 WHO 3) | (4) |
| *PIK3CA/5290* | 3q26.32 | E542K  E545K  H1047R  R108H | 7% (150) | (39) |
|  |  |  | 3% (34 WHO 1 & 11 WHO 2) | (12) |
|  |  |  | 0% (15 WHO 1 & 3 WHO 2) | (10) |
|  |  |  | 1% (82 WHO 1 & 11 WHO 2) | (32) |
| *POLR2A/5430* | 17p13.1 | Q403K  L438_H439del | 6% (775) | (5) |
| *PTEN/5728* | 10q23.31 | C136Y | 1% (55 WHO 1 & 10 WHO 2 & 10 WHO 3) | (40)  (47) |
| *SEMA4D/10507* | 9q22.2 | A327T  R713K  H806Q | 11% (9 WHO 3) | (4) |
| *SMARCA4/6597* | 19p13.2 | G1644S | 6% (15 WHO 1 & 3 WHO 2) | (10) |
| *SMARCB1/6598* | 22q11.23 | R377H | 11% (15 WHO 1 & 3 WHO 2) | (10) |
| *SMARCE1/6605* | 17q21.2 |  | 75% (12 **clear cell** meningiomas) | (50) |
| *SMO/6608* | 7q32.1 | W535L  L412F  R168H | 6% WHO I (47 WHO 1, 15 WHO 2 & 3 WHO 3) | (15) |
|  |  |  | 6% (34 WHO 1 &11 WHO 2) | (12) |
|  |  |  | 32% WHO I (69 WHO 1 & 9 WHO 2 **olfatory** groove meningiomas) | (7) |
|  |  |  | 8% (84 WHO 1 & 6 WHO 2 & 2 WHO 3) | (7) |
|  |  |  | 11% (15 WHO 1 & 3 WHO 2) | (10) |
| *SPOCD1/10853* | 1p35.2 | H468N  R436W | 13% (16 **secretory** meningiomas) | (30) |
| *TERT/7015* | 5p15.33 | C250T  A279T  C228T | 7% (85) | (33) |
|  |  |  | 2% WHO 1, 6% WHO 2 & 20% WHO 3 (119 WHO 1 88 WHO 2 & 45 WHO 3) | (34) |
|  |  |  | 14% (57 WHO 3) | (35) |
|  |  |  | 7% (87 WHO 2/3) | (36) |
|  |  |  | 6% (128) | (37) |
|  |  |  | 8% (532) | (38) |
|  |  |  | 6% (15 WHO 1 & 3 WHO 2) | (10) |
| *TP53/7157* | 17p13.1 | ^c^17 mutations | 68% (48^d^) | (51) |
| *TRAF7/84231* | 16p13.3 | ^e^23 mutations | 38% WHO 1, 18% WHO 2 (34 WHO 1 &11 WHO 2) 100% (3/3 (100%) **secretory** meningioma) | (12) |
|  |  |  | 11% (9 WHO 3) | (4) |
|  |  |  | 93% (16 **secretory** meningiomas) | (30) |
|  |  |  | 2% (84 WHO 1 & 6 WHO 2 & 2 WHO 3)  (100% of TRAF7 mutated meningiomas were **secretory** meningiomas) | (13) |

^a^Total number of tumors evaluated and mutations identified. ^b^The *NF2* gene was affected by 80 different mutations (reference between brackets for each mutation): L14fs K17_M29del V24→29Stop V24fs E32Stop E38Stop W41fs K44X D45fs L46→47Stop R52fs R57Stop T59fs F62LfsX61 363+1G>C Q65Stop Q65fs L68fs K76fs D78→83Stop H84fs E89X K99fs E103fs Q111Stop 115-1G>A F119del L127X Y144X V146I S156 R L163→173Stop L163CfsX46 (24171707) E167 D W191fs Y207fs L208P I210fs E212 Stop Q212X Y217X V219M_ 2 Patients Y221Stop A221V K228fs N248fs R249fs T251fs K253→260Stop F256fs R262X E270D D281MfsX15 F285LfsX11 K289X Q298→308Stop Q319X K332fs R341Stop Q389RfsX37 E392X K396fs L397→425Stop Q400X Q411fs R418→425Stop E422X E422→425Stop M426fs S444→454Stop E445→454 StopR447c→484Stop A451fs Q453X Q459X E460X A464fs, unknown F507fs 599 + 1G > T. ^c^The *TP53* gene was affected by 17 different mutations: E62del,Fr P64A E68ins A70ins P71S R72P V73M P77S P82del P82ins P82L W91R P92S Q100H Q104Q G105S G108R. ^d^Only exon 4 of the *TP53* gene was assessed for the presence of *TP53* mutations. ^e^The TRAF7 gene was affected by 23 different mutations: T145M F337S E353 ins FRRDASQ384E C388Y G390E T391I N520S N520H N520T H521N G536S G559V S561N Y577D L580del K615E Y621N Q637H R641C R641P R641H R653Q.

**Supplementary Table 3.** Frequency and AAF of non-synonymous genetic variants in paired peripheral blood (PB) and tumor samples from three different WHO grade 1 meningioma patients.

| Type of mutation | Altered gene | Chromosome location | AAF genetic variants | | | | | | | |
| --- | --- | --- | --- | --- | --- | --- | --- | --- | --- | --- |
|  |  |  | Peripheral blood | | | | Tumor tissue | | | |
|  |  |  | (n=3) | Patient 1 | Patient 2 | Patient 3 | (n=3) | Patient 1 | Patient 2 | Patient 3 |
| Germinal | *PNMA6A* | 153074186 | 1/3 | 0 | 0 | 0.96 | 1/3 | 0 | 0 | 0.98 |
| Somatic | *NF2* | 29639100 | 0/3 | 0 | 0 | 0 | 1/3 | 0 | 0.26 | 0 |
|  |  | 29655628 | 0/3 | 0 | 0 | 0 | 1/3 | 0 | 0 | 0.71 |
|  | *POLR2A* | 7498082 | 0/3 | 0 | 0 | 0 | 1/3 | 0.39 | 0 | 0 |
|  | *PTEN* | 87863951 | 0/3 | 0 | 0 | 0 | 1/3 | 0 | 0 | 0.33 |

## Supplementary Figures


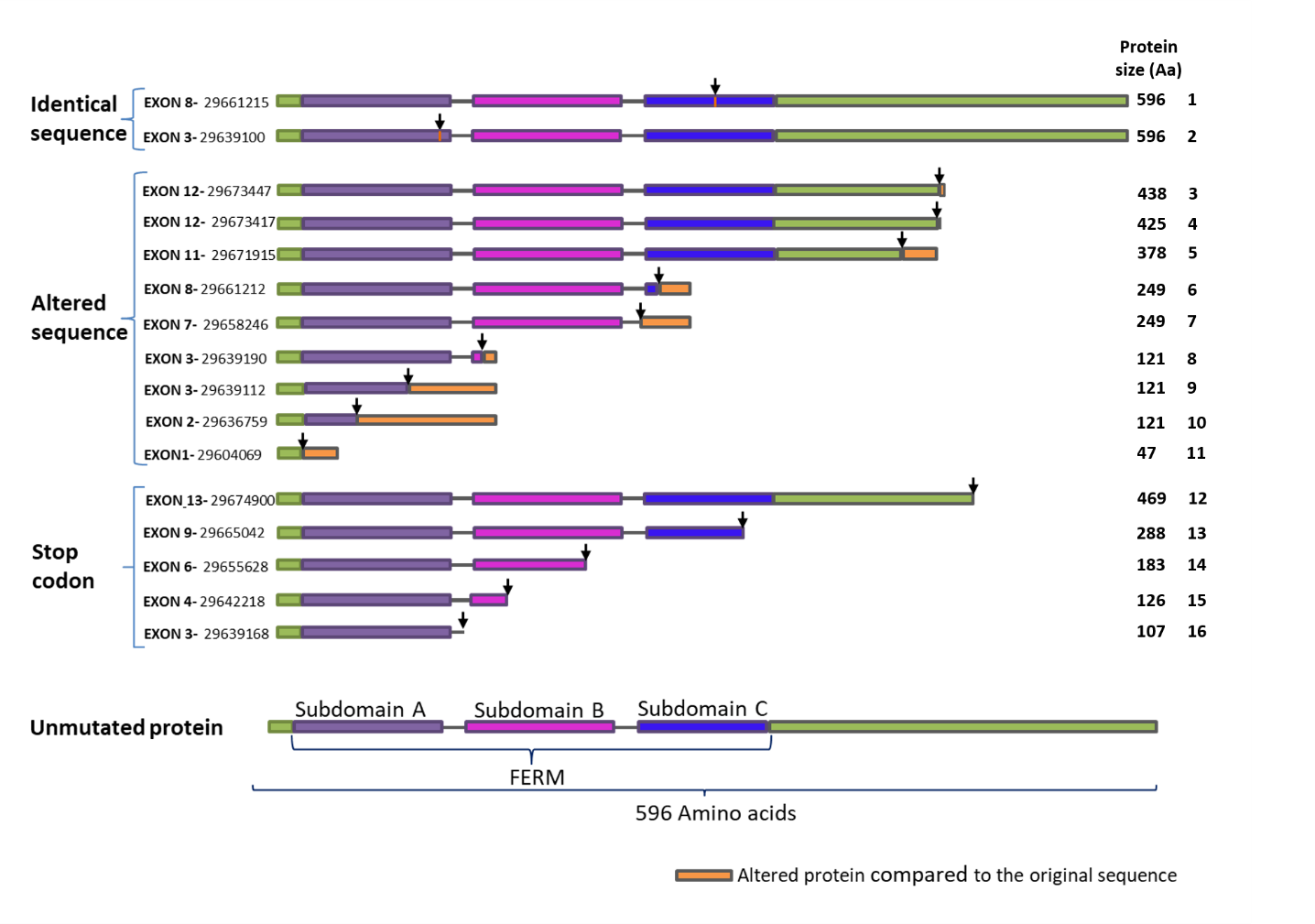


**Supplementary Figure 1.** **Graphical representation of the (truncated) protein sequence resulting from the 16 different non-synonymous genetic variants involving the *NF2* gene sequence identified in 15/32 WHO grade 1 meningiomas (one of which displayed two different variants) investigated.** Black arrows indicate the location of the predicted change at the protein level as a consequence of the genetic variants identified. The protein fragments depicted in orange reflect the new (altered) protein sequence that would emerge due to the distinct genetic variants that might affect or not the reading frame of the *NF2* gene in the presence vs absence of a stop codon. The normal protein sequence is shown at the bottom of the graphic.

## References:

1. Ostrom QT, Cioffi G, Gittleman H, Patil N, Waite K, Kruchko C, Barnholtz-Sloan JS. CBTRUS Statistical Report: Primary Brain and Other Central Nervous System Tumors Diagnosed in the United States in 2012–2016. *Neuro Oncol* (2019) **21**:v1–v100. doi:10.1093/neuonc/noz150

2. Linsler S, Kraemer D, Driess C, Oertel J, Kammers K, Rahnenfuhrer J, Ketter R, Urbschat S. Molecular biological determinations of meningioma progression and recurrence. *PLoS One* (2014) **9**:e94987. Available at: http://www.ncbi.nlm.nih.gov/entrez/query.fcgi?cmd=Retrieve&db=PubMed&dopt=Citation&list_uids=24722350

3. Domingues PH, Sousa P, Otero Á, Gonçalves JM, Ruiz L, de Oliveira C, Lopes MC, Orfao A, Tabernero MD. Proposal for a new risk stratification classification for meningioma based on patient age, WHO tumor grade, size, localization, and karyotype. *Neuro Oncol* (2014) **16**:735–47. doi:10.1093/neuonc/not325

4. Zhang X, Jia H, Lu Y, Dong C, Hou J, Wang Z, Wang F, Zhong H, Wang L, Wang K. Exome sequencing on malignant meningiomas identified mutations in neurofibromatosis type 2 (NF2) and meningioma 1 (MN1) genes. *Discov Med* (2014) **18**:301–311. Available at: http://www.ncbi.nlm.nih.gov/pubmed/25549701

5. Clark VE, Harmancl AS, Bai H, Youngblood MW, Lee TI, Baranoski JF, Ercan-Sencicek AG, Abraham BJ, Weintraub AS, Hnisz D, et al. Recurrent somatic mutations in POLR2A define a distinct subset of meningiomas. *Nat Genet* (2016) **48**:1253–1259. doi:10.1038/ng.3651

6. Shankar GM, Abedalthagafi M, Vaubel RA, Merrill PH, Nayyar N, Gill CM, Brewster R, Bi WL, Agarwalla PK, Thorner AR, et al. Germline and somatic BAP1 mutations in high-grade rhabdoid meningiomas. *Neuro Oncol* (2017) **19**:535–545. doi:10.1093/neuonc/now235

7. Boetto J, Bielle F, Sanson M, Peyre M, Kalamarides M. SMO mutation status defines a distinct and frequent molecular subgroup in olfactory groove meningiomas. *Neuro Oncol* (2017) **19**:345–351. doi:10.1093/neuonc/now276

8. Szijan I, Rochefort D, Bruder C, Surace E, Machiavelli G, Dalamon V, Cotignola J, Ferreiro V, Campero A, Basso A, et al. NF2 tumor suppressor gene: a comprehensive and efficient detection of somatic mutations by denaturing HPLC and microarray-CGH. *Neuromolecular Med* (2003) **3**:41–52. doi:10.1385/NMM:3:1:41

9. Tabernero MD, Jara-Acevedo M, Nieto AB, Caballero AR, Otero A, Sousa P, Goncalves J, Domingues PH, Orfao A, Otero Á, et al. Association between mutation of the NF2 gene and monosomy 22 in menopausal women with sporadic meningiomas. *BMC Med Genet* (2013) **14**:114. doi:10.1186/1471-2350-14-114

10. Jungwirth G, Warta R, Beynon C, Sahm F, von Deimling A, Unterberg A, Herold-Mende C, Jungk C. Intraventricular meningiomas frequently harbor NF2 mutations but lack common genetic alterations in TRAF7, AKT1, SMO, KLF4, PIK3CA, and TERT. *Acta Neuropathol Commun* (2019) **7**:140. doi:10.1186/s40478-019-0793-4

11. Bi WL, Greenwald NF, Abedalthagafi M, Wala J, Gibson WJ, Agarwalla PK, Horowitz P, Schumacher SE, Esaulova E, Mei Y, et al. Genomic landscape of high-grade meningiomas. *npj Genomic Med* (2017) **2**:1–14. doi:10.1038/s41525-017-0014-7

12. Clark VE, Erson-Omay EZ, Serin A, Yin J, Cotney J, Ozduman K, Avsar T, Li J, Murray PB, Henegariu O, et al. Genomic analysis of non-NF2 meningiomas reveals mutations in TRAF7, KLF4, AKT1, and SMO. *Science* (2013) **339**:1077–80. doi:10.1126/science.1233009

13. Hao S, Huang G, Feng J, Li D, Wang K, Wang L, Wu Z, Wan H, Zhang L, Zhang J. Non-NF2 mutations have a key effect on inhibitory immune checkpoints and tumor pathogenesis in skull base meningiomas. *J Neurooncol* (2019) **144**:11–20. doi:10.1007/s11060-019-03198-9

14. Bi WL, Abedalthagafi M, Horowitz P, Agarwalla PK, Mei Y, Aizer AA, Brewster R, Dunn GP, Al-Mefty O, Alexander BM, et al. Genomic landscape of intracranial meningiomas. *J Neurosurg* (2016) **125**:525–535. doi:10.3171/2015.6.JNS15591

15. Brastianos PK, Horowitz PM, Santagata S, Jones RT, McKenna A, Getz G, Ligon KL, Palescandolo E, Van Hummelen P, Ducar MD, et al. Genomic sequencing of meningiomas identifies oncogenic SMO and AKT1 mutations. *Nat Genet* (2013) **45**:285–9. doi:10.1038/ng.2526

16. Tabernero MD, Maíllo A, Nieto AB, Diez-Tascón C, Lara M, Sousa P, Otero A, Castrillo A, Patino-Alonso M del C, Espinosa A, et al. Delineation of commonly deleted chromosomal regions in meningiomas by high-density single nucleotide polymorphism genotyping arrays. *Genes Chromosomes Cancer* (2012) **51**:606–17. doi:10.1002/gcc.21948

17. Chen F, Xiang CX, Zhou Y, Ao XS, Zhou DQ, Peng P, Zhang HQ, Liu HD, Huang X. Gene expression profile for predicting survival of patients with meningioma. *Int J Oncol* (2015) **46**:791–797. doi:10.3892/ijo.2014.2779

18. Chen S, Zhou Y, Chen Y, Gu J. fastp: an ultra-fast all-in-one FASTQ preprocessor. *Bioinformatics* (2018) **34**:i884–i890. doi:10.1093/bioinformatics/bty560

19. Van der Auwera GA, Carneiro MO, Hartl C, Poplin R, Del Angel G, Levy-Moonshine A, Jordan T, Shakir K, Roazen D, Thibault J, et al. From FastQ data to high confidence variant calls: the Genome Analysis Toolkit best practices pipeline. *Curr Protoc Bioinforma* (2013) **43**:11 10 1-33. doi:10.1002/0471250953.bi1110s43

20. Li H. A statistical framework for SNP calling, mutation discovery, association mapping and population genetical parameter estimation from sequencing data. *Bioinformatics* (2011) **27**:2987–2993. doi:10.1093/bioinformatics/btr509

21. De Baets G, Van Durme J, Reumers J, Maurer-Stroh S, Vanhee P, Dopazo J, Schymkowitz J, Rousseau F. SNPeffect 4.0: on-line prediction of molecular and structural effects of protein-coding variants. *Nucleic Acids Res* (2012) **40**:D935-9. doi:10.1093/nar/gkr996

22. Wang K, Li M, Hakonarson H. ANNOVAR: functional annotation of genetic variants from high-throughput sequencing data. *Nucleic Acids Res* (2010) **38**:e164. doi:10.1093/nar/gkq603

23. Lek M, Karczewski KJ, Minikel E V, Samocha KE, Banks E, Fennell T, O’Donnell-Luria AH, Ware JS, Hill AJ, Cummings BB, et al. Analysis of protein-coding genetic variation in 60,706 humans. *Nature* (2016) **536**:285–291. doi:10.1038/nature19057

24. 1000 Genomes Project Consortium, Auton A, Brooks LD, Durbin RM, Garrison EP, Kang HM, Korbel JO, Marchini JL, McCarthy S, McVean GA, et al. A global reference for human genetic variation. *Nature* (2015) **526**:68–74. doi:10.1038/nature15393

25. Konrad J. Karczewski, Laurent C. Francioli, Grace Tiao, Beryl B Jessica C, Alföldi 2,4, Ryan L. Collins1,4,5, Kristen M. Laricchia1,2, Andrea Ganna1,2,6, Daniel QW, P. Birnbaum1 Laura D. Gauthier7 2, 5, Matthew Solomonson1,2, Nicholas A. HB, Watts1 Daniel Rhodes8 2, 2, Eleina M. England1,2, Eleanor G. Seaby1,2, Jack MS-B, A. Kosmicki1 4, Raymond K. Walters1,2,9, Katherine Tashman1,2,9, Yossi Farjoun7 2, et al. Variation across 141,456 human exomes and genomes reveals the spectrum of loss-offunction intolerance across human protein-coding genes . (2019)

26. Domingues P, González-Tablas M, Otero Á, Pascual D, Ruiz L, Miranda D, Sousa P, Gonçalves JM, Lopes MC, Orfao A, et al. Genetic/molecular alterations of meningiomas and the signaling pathways targeted. *Oncotarget* (2015) **6**:10671–10688. doi:10.18632/oncotarget.3870

27. Hansson CM, Buckley PG, Grigelioniene G, Piotrowski A, Hellstrom AR, Mantripragada K, Jarbo C, Mathiesen T, Dumanski JP. Comprehensive genetic and epigenetic analysis of sporadic meningioma for macro-mutations on 22q and micro-mutations within the NF2 locus. *BMC Genomics* (2007) **8**:16. doi:10.1186/1471-2164-8-16

28. Kim JH, Kim IS, Kwon SY, Jang BC, Suh S Il, Shin DH, Jeon CH, Son EI, Kim SP. Mutational analysis of the NF2 gene in sporadic meningiomas by denaturing high-performance liquid chromatography. *Int J Mol Med* (2006) **18**:27–32. Available at: http://www.ncbi.nlm.nih.gov/pubmed/16786152

29. Hartmann C, Sieberns J, Gehlhaar C, Simon M, Paulus W, von Deimling A. NF2 mutations in secretory and other rare variants of meningiomas. *Brain Pathol* (2006) **16**:15–19. Available at: http://www.ncbi.nlm.nih.gov/entrez/query.fcgi?cmd=Retrieve&db=PubMed&dopt=Citation&list_uids=16612978

30. Reuss DE, Piro RM, Jones DT, Simon M, Ketter R, Kool M, Becker A, Sahm F, Pusch S, Meyer J, et al. Secretory meningiomas are defined by combined KLF4 K409Q and TRAF7 mutations. *Acta Neuropathol* (2013) **125**:351–358. doi:10.1007/s00401-013-1093-x

31. Sahm F, Bissel J, Koelsche C, Schweizer L, Capper D, Reuss D, Bohmer K, Lass U, Gock T, Kalis K, et al. AKT1E17K mutations cluster with meningothelial and transitional meningiomas and can be detected by SFRP1 immunohistochemistry. *Acta Neuropathol* (2013) **126**:757–762. doi:10.1007/s00401-013-1187-5

32. Yesiloz U, Kirches E, Hartmann C, Scholz J, Kropf S, Sahm F, Nakamura M, Mawrin C. Frequent AKT1E17K mutations in skull base meningiomas are associated with mTOR and ERK1/2 activation and reduced time to tumor recurrence. *Neuro Oncol* (2017) **19**:1088–1096. doi:10.1093/neuonc/nox018

33. Goutagny S, Nault JC, Mallet M, Henin D, Rossi JZ, Kalamarides M. High incidence of activating TERT promoter mutations in meningiomas undergoing malignant progression. *Brain Pathol* (2014) **24**:184–189. doi:10.1111/bpa.12110

34. Sahm F, Schrimpf D, Olar A, Koelsche C, Reuss D, Bissel J, Kratz A, Capper D, Schefzyk S, Hielscher T, et al. TERT Promoter Mutations and Risk of Recurrence in Meningioma. *J Natl Cancer Inst* (2016) **108**: doi:10.1093/jnci/djv377

35. Peyre M, Gauchotte G, Giry M, Froehlich S, Pallud J, Graillon T, Bielle F, Cazals-Hatem D, Varlet P, Figarella-Branger D, et al. De novo and secondary anaplastic meningiomas: a study of clinical and histomolecular prognostic factors. *Neuro Oncol* (2018) **20**:1113–1121. doi:10.1093/neuonc/nox231

36. Biczok A, Kraus T, Suchorska B, Terpolilli NA, Thorsteinsdottir J, Giese A, Tonn JC, Schichor C. TERT promoter mutation is associated with worse prognosis in WHO grade II and III meningiomas. *J Neurooncol* (2018) **139**:671–678. doi:10.1007/s11060-018-2912-7

37. Spiegl-Kreinecker S, Lotsch D, Neumayer K, Kastler L, Gojo J, Pirker C, Pichler J, Weis S, Kumar R, Webersinke G, et al. TERT promoter mutations are associated with poor prognosis and cell immortalization in meningioma. *Neuro Oncol* (2018) **20**:1584–1593. doi:10.1093/neuonc/noy104

38. Lu VM, Goyal A, Lee A, Jentoft M, Quinones-Hinojosa A, Chaichana KL. The prognostic significance of TERT promoter mutations in meningioma: a systematic review and meta-analysis. *J Neurooncol* (2019) **142**:1–10. doi:10.1007/s11060-018-03067-x

39. Abedalthagafi M, Bi WL, Aizer AA, Merrill PH, Brewster R, Agarwalla PK, Listewnik ML, Dias-Santagata D, Thorner AR, Van Hummelen P, et al. Oncogenic PI3K mutations are as common as AKT1 and SMO mutations in meningioma. *Neuro Oncol* (2016) **18**:649–55. doi:10.1093/neuonc/nov316

40. Peters N, Wellenreuther R, Rollbrocker B, Hayashi Y, Meyer-Puttlitz B, Duerr EM, Lenartz D, Marsh DJ, Schramm J, Wiestler OD, et al. Analysis of the PTEN gene in human meningiomas. *Neuropathol Appl Neurobiol* (1998) **24**:3–8. doi:10.1046/j.1365-2990.1998.00093.x

41. Lorenz J, Rothhammer-Hampl T, Zoubaa S, Bumes E, Pukrop T, Kölbl O, Corbacioglu S, Schmidt NO, Proescholdt M, Hau P, et al. A comprehensive DNA panel next generation sequencing approach supporting diagnostics and therapy prediction in neurooncology. *Acta Neuropathol Commun* (2020) **8**:124. doi:10.1186/s40478-020-01000-w

42. Muñoz-González JI, Álvarez-Twose I, Jara-Acevedo M, Henriques A, Viñas E, Prieto C, Sánchez-Muñoz L, Caldas C, Mayado A, Matito A, et al. Frequency and prognostic impact of KIT and other genetic variants in indolent systemic mastocytosis. *Blood* (2019) **134**:456–468. doi:10.1182/blood.2018886507

43. Muñoz-González JI, Jara-Acevedo M, Alvarez-Twose I, Merker JD, Teodosio C, Hou Y, Henriques A, Roskin KM, Sanchez-Muñoz L, Tsai AG, et al. Impact of somatic and germline mutations on the outcome of systemic mastocytosis. *Blood Adv* (2018) **2**:2814–2828. doi:10.1182/bloodadvances.2018020628

44. Díaz-Casado E, Gómez-Nieto R, de Pereda JM, Muñoz LJ, Jara-Acevedo M, López DE. Analysis of gene variants in the GASH/Sal model of epilepsy. *PLoS One* (2020) **15**:e0229953. doi:10.1371/journal.pone.0229953

45. Sun Z, Lu J, Wu M, Li M, Bai L, Shi Z, Hao L, Wu Y. Deficiency of PTEN leads to aberrant chromosome segregation through downregulation of MAD2. *Mol Med Rep* (2019) doi:10.3892/mmr.2019.10668

46. Paganin M, Grillo MF, Silvestri D, Scapinello G, Buldini B, Cazzaniga G, Biondi A, Valsecchi MG, Conter V, te Kronnie G, et al. The presence of mutated and deleted PTEN is associated with an increased risk of relapse in childhood T cell acute lymphoblastic leukaemia treated with AIEOP-BFM ALL protocols. *Br J Haematol* (2018) **182**:705–711. doi:10.1111/bjh.15449

47. Joachim T, Ram Z, Rappaport ZH, Simon M, Schramm J, Wiestler OD, von Deimling A. Comparative analysis of the NF2, TP53, PTEN, KRAS, NRAS and HRAS genes in sporadic and radiation-induced human meningiomas. *Int J Cancer* (2001) **94**:218–221. Available at: http://www.ncbi.nlm.nih.gov/entrez/query.fcgi?cmd=Retrieve&db=PubMed&dopt=Citation&list_uids=11668501

48. Martinez-Glez V, Bello MJ, Franco-Hernandez C, De Campos JM, Isla A, Vaquero J, Rey JA. Mutational analysis of the DAL-1/4.1B tumour-suppressor gene locus in meningiomas. *Int J Mol Med* (2005) **16**:771–774. Available at: http://www.ncbi.nlm.nih.gov/entrez/query.fcgi?cmd=Retrieve&db=PubMed&dopt=Citation&list_uids=16142420

49. Dobbins SE, Broderick P, Melin B, Feychting M, Johansen C, Andersson U, Brannstrom T, Schramm J, Olver B, Lloyd A, et al. Common variation at 10p12.31 near MLLT10 influences meningioma risk. *Nat Genet* (2011) **43**:825–827. doi:ng.879 [pii]10.1038/ng.879

50. Smith MJ, Ahn S, Lee JI, Bulman M, Plessis DD, Suh YL. SMARCE1 mutation screening in classification of clear cell meningiomas. *Histopathology* (2017) **70**:814–820. doi:10.1111/his.13135

51. Bukovac A, Kafka A, Hrascan R, Vladusic T, Pecina-Slaus N. Nucleotide variations of TP53 exon 4 found in intracranial meningioma and in silico prediction of their significance. *Mol Clin Oncol* (2019) **11**:563–572. doi:10.3892/mco.2019.1936
